# VMP1 attenuates ferroptosis and mitochondrial dysfunction in nucleus pulposus cells through the PINK1/Parkin-mediated mitophagy pathway

**DOI:** 10.1186/s13018-025-06033-2

**Published:** 2025-07-08

**Authors:** Yang Zhang, Yucheng Gao, Shuanggong Liu, Guowei Yang, Yijun Rong, Dongjin Wu, Zengxin Gao

**Affiliations:** 1 Department of Spinal Surgery, Zhongda Hospital, College of Medicine, Southeast University, NO.87 Ding Jia Qiao, Nanjing, Jiangsu 210009 People’s Republic of China; 2 Department of Orthopaedics, The Second Hospital of Shandong University, 247 Beiyuan Street, Jinan, Shandong 250033 People’s Republic of China

**Keywords:** IVDD, VMP1, Ferroptosis, Mitochondrial dysfunction, Mitophagy

## Abstract

**Background:**

Intervertebral disc degeneration (IVDD) is a multifactorial disorder and a leading contributor to chronic low back pain (LBP), highlighting the need for novel therapeutic strategies. Recent studies indicate that ferroptosis, driven by oxidative stress, plays a key role in the loss of nucleus pulposus cells (NPCs) during IVDD. Vacuole membrane protein 1 (VMP1), a membrane-associated regulator of autophagy, is known to influence various cellular processes. However, its role in IVDD remains unclear. This study investigates the function of VMP1 in IVDD and the mechanisms involved.

**Methods:**

We established a rat model of IVDD to investigate the correlation between VMP1 expression and ferroptosis during IVDD progression. In vitro, a ferroptosis model of NPCs was induced using tert-butyl hydroperoxide (TBHP) to examine the effects of VMP1 knockdown on NPC apoptosis, extracellular matrix (ECM) degradation, ferroptosis, PINK1/Parkin-dependent mitophagy, and mitochondrial function. Furthermore, cyclosporin A (CsA), a mitophagy inhibitor, was employed to explore the role and potential mechanisms of VMP1 overexpression in regulating PINK1/Parkin-mediated mitophagy, mitochondrial function, and ferroptosis.

**Results:**

In this study, we observed a significant downregulation of VMP1 expression in a rat model of IVDD, which was accompanied by the occurrence of ferroptosis. Subsequent experiments revealed that VMP1 knockdown aggravated apoptosis and ECM degradation in NPCs. Furthermore, we demonstrated that VMP1 silencing promoted ferroptosis, inhibited PINK1/Parkin-dependent mitophagy, and impaired mitochondrial function in NPCs. In contrast, VMP1 overexpression enhanced PINK1/Parkin-mediated mitophagy, mitigated mitochondrial dysfunction, and suppressed ferroptosis. Notably, these protective effects were abolished by treatment with CsA.

**Conclusions:**

This study demonstrates that VMP1 alleviates IVDD by inhibiting ferroptosis and mitochondrial dysfunction in NPCs, a protective effect mediated through the promotion of PINK1/Parkin-dependent mitophagy. Our study underscores the pivotal role of VMP1 in coordinating mitophagy and ferroptosis during IVDD pathogenesis, identifying VMP1 as a potential therapeutic target for IVDD treatment.

**Graphical Abstract:**

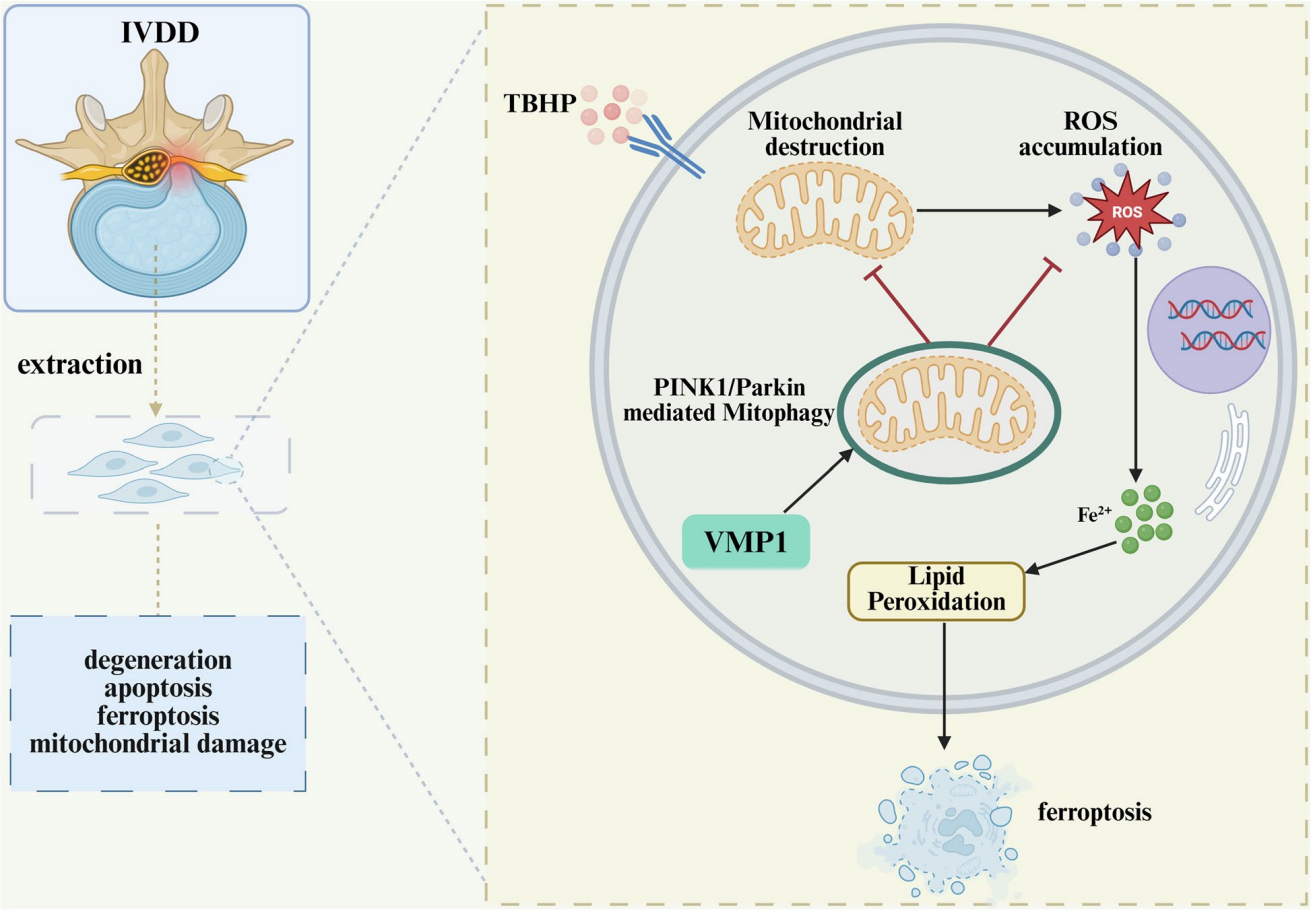

**Supplementary Information:**

The online version contains supplementary material available at 10.1186/s13018-025-06033-2.

## Introduction

Intervertebral disc degeneration (IVDD) is a highly prevalent musculoskeletal disorder and a leading contributor to chronic low back pain (LBP), affecting a substantial portion of the global population and imposing a significant socioeconomic burden [1, 2]. With up to 84% of people experiencing low back pain, IVDD accounts for over 40% of cases and is increasingly prevalent in the aging population, often leading to mobility loss or severe neurological deficits [3, 4]. Anatomically, the intervertebral disc (IVD) is composed of the nucleus pulposus (NP), annulus fibrosus (AF), and cartilaginous endplates (CEPs), which together maintain spinal flexibility and mechanical integrity [5]. Among them, the NP plays a central role in absorbing axial loads and maintaining disc elasticity through the secretion of proteoglycans and type II collagen by nucleus pulposus cells (NPCs) [6]. However, due to its avascular nature and dependence on limited nutrient diffusion from adjacent endplates, the IVD is highly susceptible to metabolic stress [7]. Under pathological stimuli such as aging, mechanical overload, and unhealthy lifestyles, the NP undergoes progressive degeneration characterized by chronic inflammation, oxidative stress, mitochondrial dysfunction, extracellular matrix (ECM) degradation, and cell death [8, 9]. These pathological changes ultimately lead to disc dehydration, reduced disc height, and compromised biomechanical function, thereby accelerating IVDD progression. Current research efforts have focused on elucidating the molecular mechanisms underlying IVDD to identify potential therapeutic targets for improved treatment strategies.

Programmed cell death (PCD) is a tightly regulated biological process essential for preserving organismal homeostasis. Dysregulation of PCD has been associated with the onset and progression of numerous pathological condition [10, 11]. Ferroptosis, a form of PCD, differs from necrosis, apoptosis, and pyroptosis both in its morphology and pathological processes, and is considered a novel type of cell death [12]. Ferroptosis is a form of regulated cell death driven by iron-dependent oxidative stress, primarily mediated through Fenton reactions and lipid peroxidation catalyzed by intracellular iron or lipoxygenases [13]. This process is characterized by depletion of glutathione (GSH) and inactivation of the glutathione peroxidase 4 (GPX4)-centered antioxidant defense system. As a result, cells exhibit hallmark features such as increased mitochondrial membrane density, excessive accumulation of lipid peroxidation products and reactive oxygen species (ROS) related to iron metabolism, along with distinct morphological changes including mitochondrial cristae collapse and outer membrane rupture [14–16]. Key regulators of ferroptosis, including GPX4 and ferritin heavy chain (FTH), confer antiferroptotic protection by reducing lipid hydroperoxides and modulating intracellular iron homeostasis, respectively [17, 18]. Emerging evidence indicates that iron overload impairs mitochondrial oxidative phosphorylation in NP and AF cells, leading to excessive ROS generation and downregulation of GPX4, thereby contributing to the progression of IVDD [19]. Although the precise role of ferroptosis in IVDD pathogenesis has yet to be fully elucidated, its regulatory network offers promising therapeutic targets for mitigating disc degeneration.

Mitophagy, a specific type of autophagy, plays a critical role in maintaining mitochondrial integrity and cellular homeostasis by selectively eliminating damaged or dysfunctional mitochondria [20]. The PINK1-mediated mitophagy pathway, a key mechanism in mammals, is vital in regulating various immune and inflammatory conditions. PINK1 activates Parkin to target a range of mitochondrial proteins, such as NDP52, OPTN, and p62, and interacts with LC3 to facilitate the transport of impaired mitochondria into autophagosomes [21]. This process can prevent the accumulation of ROS, alleviate mitochondrial dysfunction, mitigate oxidative stress, and inhibit PCD. Recent studies have identified a critical association between mitophagy and ferroptosis, underscoring the impact of mitochondrial dysfunction in oxidative stress-related disorders [22]. Mitophagy appears to suppress ferroptosis by clearing impaired mitochondria, limiting ROS buildup, preserving iron homeostasis, and influencing lipid metabolism [23]. The reciprocal regulation and molecular crosstalk between these two processes have been observed in multiple conditions, including IVDD [24]. PINK1/Parkin-mediated mitophagy may exert a protective effect in the pathogenesis of IVDD by modulating oxidative stress-induced ferroptosis in NPCs.

Vacuole membrane protein 1 (VMP1) is an integral endoplasmic reticulum (ER) transmembrane protein originally identified in acute pancreatitis and is now recognized as a key regulator of autophagy and related cellular processes [25, 26]. VMP1 and its paralog protein TMEM41B (transmembrane protein 41B), both ER-resident phospholipid scramblases essential for autophagy, cooperate to equilibrate phospholipid distribution between the outer and inner leaflets of the ER and phagophore membranes, thereby correcting autophagic defects and maintaining cellular homeostasis [27]. VMP1 has roles that go beyond its involvement in autophagy, with growing evidence indicating its importance in regulating endoplasmic reticulum calcium homeostasis—an essential factor for proper protein folding, intracellular signaling, and cellular responses to viral infection [28, 29]. Recent research has also implicated VMP1 in cellular defense mechanisms against viral infections and in lipid droplet (LD) metabolism. Aberrant expression or dysfunction of VMP1 has been reported in various pathological conditions—including neurodegenerative disorders like Parkinson’s disease (PD), as well as pancreatitis, hepatitis, and cancer—highlighting its potential as a promising therapeutic target [30–32]. However, the physiological and pathological roles of VMP1 in IVDD remain unexplored.

This study aims to investigate the role of VMP1 in IVDD progression and to elucidate the interplay between PINK1-dependent mitophagy and ferroptosis and mitochondrial dysfunction induced by oxidative stress, offering novel perspectives for IVDD treatment.

## Materials and methods

### Animal experiments and ethics statement

The animal experiments conducted in this study received approval from the Ethics Committee of the Medical School of Southeast University (Ethics Approval Number: SEU-IACUC-20250220008) and were performed in compliance with the"Guidelines for the Welfare and Ethical Review of Laboratory Animals"(GB/T 35892–2018, National Standards of the People's Republic of China). A classic and extensively validated rat model of IVDD was established via fine-needle puncture of the coccygeal discs, a method commonly employed to mimic the progressive structural and functional deterioration observed in human IVDD [33, 34]. Male Sprague–Dawley (SD) rats, aged eight weeks and weighing approximately 230 ± 20 g. Anesthesia was induced using 3% pentobarbital sodium (1 ml/kg), and manual palpation was performed to identify an appropriate intervertebral disc level for puncture, specifically targeting the third and fourth coccygeal discs. Following disinfection with iodine, a dorsal midline skin incision was made to expose the outer AF. A 21G needle was carefully inserted through the AF, advanced into the NP, and further penetrated the contralateral AF. The needle was rotated 360° and held in place for 30 s before removal. The control group did not receive any intervention. Muscle layers were closed using 3–0 silk sutures, and skin edges were sutured with 4–0 nylon. Six weeks postoperatively, the rats were euthanized, and tissue specimens were harvested for histological analysis using hematoxylin and eosin (H&E) and Safranin O/fast green staining, while immunohistochemical staining was performed to evaluate the expression of VMP1.

### Isolation and culture of primary rat NPCs

NPCs were isolated from the coccygeal intervertebral discs of 8-week-old male SD rats. The harvested nucleus pulposus tissue was transferred to a sterile laminar flow hood, where it was rinsed 3–5 times with sterile phosphate-buffered saline (PBS; Servicebio, Wuhan, China) to eliminate residual blood. The tissue was then finely minced using ophthalmic scissors and washed an additional three times with PBS. Enzymatic digestion was performed sequentially: initially with 0.25% trypsin–EDTA (Servicebio, China) for 5 min, followed by overnight incubation at 37 °C with 0.2% type II collagenase (Solarbio, Beijing, China). The digested suspension was passed through a 200-mesh filter to obtain a single-cell suspension of NPCs. After centrifugation, the supernatant was discarded and the cell pellet was resuspended in DMEM/F12 medium (Basalmedia, Shanghai, China) supplemented with 10% fetal bovine serum (ViviCell, Shanghai, China) and 1% penicillin–streptomycin (Gibco, USA). Cells were maintained at 37 °C in a humidified incubator with 5% CO₂. The culture medium was changed every three days. Cells at passages 1 to 3 (P1–P3) were used for subsequent experiments once they reached approximately 80% confluence. To establish an in vitro ferroptosis model relevant to IVDD, NPCs were treated with 100 μM tert-butyl hydroperoxide (TBHP; Aladdin, Shanghai, China) for 4 h [35, 36]. Subsequently, TBHP-containing medium was replaced with fresh complete medium for further assays. In this context, untreated NPCs were designated as the control group, representing physiologically normal cells, whereas TBHP-treated cells served as the in vitro ferroptosis model.

### Plasmid transfection and Lentiviral transfection

A short hairpin RNA (shRNA) vector specifically targeting VMP1 was purchased from Tsingke Biotechnology Co., Ltd. (Beijing, China). NPCs were seeded in 6-well culture plates and, upon reaching approximately 80% confluence, the growth medium was replaced. Transfection of the shRNA plasmid into NPCs was carried out using Lipo8000™ transfection reagent (Beyotime, Beijing, China), following the manufacturer’s instructions. Cells transfected with a non-targeting scrambled shRNA sequence were used as a negative control (sh-NC), which does not induce any specific gene silencing and is commonly employed to account for non-specific effects of shRNA transfection. For overexpression of VMP1, NPCs were transduced with recombinant lentiviral vectors (Lv-VMP1 or Lv-NC) obtained from Wzbio (Shandong, China). Once the cells reached approximately 80% confluence, lentiviral infection was performed at a multiplicity of infection (MOI) of 50, according to the supplier's protocol. After 24 h of incubation, cell viability exceeded 90%, at which point the medium was replaced with fresh complete culture medium for subsequent experiments.

### Extraction of total RNA and quantitative real-time PCR (qRT-PCR) analysis

Total RNA was extracted from NPCs using TRIzol reagent (Thermo Fisher Scientific, Waltham, MA, USA) in accordance with the manufacturer’s instructions. Complementary DNA (cDNA) was synthesized from the isolated RNA using a reverse transcription kit (Thermo Fisher Scientific, USA). The synthesized cDNA was subsequently diluted 1:10 prior to use in downstream applications. Quantitative real-time PCR was performed using a QuantStudio™ 5 Real-Time PCR System (Thermo Fisher Scientific, USA) to assess the mRNA expression levels of VMP1, GPX4, FTH1, LPCAT3, and ACSL4 in NPCs. GAPDH was employed as the internal reference gene. Each qPCR reaction was carried out in a total volume of 20 μL, containing 10 μL of SYBR Green Master Mix (Thermo Fisher Scientific), 0.5 μL of each primer (10 μM), 1 μL of diluted cDNA template, and 8 μL of RNase-free water. The thermal cycling protocol consisted of an initial denaturation at 95 °C for 10 min, followed by 40 cycles of 95 °C for 15 s and 60 °C for 30 s. Melting curve analysis was performed at the end of the amplification cycles to confirm the specificity of the products. The specific primer sequences used for qPCR are listed in Table 1.

**Table 1:** Primers for real-time PCR

	Primer Information (rat)
GAPDH	Forward-ACACCCACTCCTCCACCTTTG
	Reverse-TCCACCACCCTGTTGCTGTAG
VMP1	Forward-TTTCCCGAACCACCCTATCC
	Reverse-CAGACTCTGCATGTTCCAGC
GPX4	Forward-AGTTCGGGAGGCAGGAG
	Reverse-CCACGCAGCCGTTCTTA
FTH1	Forward-GCCGAGAAACTGATGAAGCTGC
	Reverse-GCACACTCCATTGCATTCAGCC
LPCAT3	Forward-GGCCTCTCAATTGCTTATTTCA
	Reverse-AGCACGACACATAGCAAGGA
ACSL4	Forward-TCCGCTTGTGACTTTAT
	Reverse-ACTTGGAGGAATGCT

### Cell viability analysis

Cell viability of NPCs was assessed using the Cell Counting Kit-8 (CCK-8; Elabscience, Wuhan, China) according to the manufacturer’s instructions. NPCs were seeded in 96-well plates at a density of approximately 5 × 10^3^ cells/well. After treatment, 10 µL of CCK-8 solution was added to each well, followed by incubation in a humidified cell culture incubator for 1 h. The absorbance at 450 nm was subsequently measured using a microplate reader to quantify cell viability.

### TUNEL assay

Apoptosis was assessed using a TUNEL staining kit (Epizyme Biotech, China). NPCs were seeded at a density of approximately 3 × 10^4^ cells per well in 12-well plates containing sterile glass slides, allowed to attach, and subsequently subjected to the indicated interventions. NPCs were first fixed in 4% paraformaldehyde at room temperature for 30 min and then permeabilized with 0.5% Triton X-100 for 5 min. Following this, 100 μL of TUNEL reaction mixture was applied, and the cells were incubated at 37 °C in the dark for 1 h. Nuclei were counterstained with DAPI. Fluorescent images were acquired using a Zeiss LSM800 laser scanning confocal microscope (Germany) to determine the proportion of TUNEL-positive cells.

### Safranin O and Alcian blue staining

The phenotypic characteristics of NPCs were assessed through Safranin O and Alcian Blue staining. NPCs were seeded at a density of approximately 6 × 10^4^ cells per well in 6-well plates and subjected to the designated treatments before being fixed with 4% paraformaldehyde at room temperature for 30 min. After fixation, the cells were rinsed three times with PBS and subsequently stained with 0.1% Safranin O solution (Solarbio, China) and 0.1% Alcian Blue solution (Solarbio, China) for 5 min. Following staining, the cells underwent three additional PBS washes and were then visualized using an optical microscope (Leica, Germany).

### SA-β-Gal staining

Senescence in NPCs was assessed using a Senescence-Associated β-Galactosidase (SA-β-Gal) Staining Kit (Beyotime, China). NPCs were cultured in 6-well plates at a density of approximately 6 × 10^4^ cells per well, exposed to the indicated treatments, and fixed with 4% paraformaldehyde at room temperature for 30 min. After fixation, cells were rinsed twice with PBS and subsequently incubated with a freshly prepared SA-β-Gal staining solution at 37 °C for 12 h. Senescent cells displaying blue staining, along with the total number of cells, were counted under a light microscope (Olympus). The percentage of SA-β-Gal–positive cells was then calculated to quantify cellular senescence.

### Immunofluorescence

NPCs were seeded into 20-mm confocal dishes and cultured in DMEM/F12 medium without serum for 24 h upon reaching approximately 80% confluency. Following treatment based on experimental grouping, cells were rinsed three times with PBS and fixed in 4% paraformaldehyde for 15 min. Permeabilization was then carried out using 0.3% Triton X-100 in PBS for 15 min, followed by additional PBS washes. To block non-specific antigen binding, cells were incubated with 10% bovine serum albumin (BSA) for 1 h at room temperature. Subsequently, cells were incubated overnight at 4 °C with the appropriate primary antibody. After three PBS washes, incubation with the corresponding fluorescent secondary antibody was performed for 1 h at room temperature in the dark. Nuclei were counterstained with DAPI for 5 min in the dark, and cells were mounted using an antifade reagent. Immunofluorescence images were acquired using a Zeiss LSM800 laser scanning confocal microscope (Zeiss, Germany), and fluorescence intensity was quantified with ImageJ software, normalized to DAPI signal levels.

### Protein extraction and western blot

In brief, following the indicated treatments, total protein was extracted from NPCs and tissue samples using RIPA lysis buffer (Beyotime, China) supplemented with protease and phosphatase inhibitors (Epizyme Biotech, China), in accordance with the manufacturers’ instructions. Protein concentrations were quantified using a BCA Protein Assay Kit (Beyotime, China). Equal amounts of protein (15 μg per sample) were resolved on 10% or 12% SDS-PAGE gels and subsequently transferred onto polyvinylidene fluoride (PVDF) membranes (Millipore, USA). The membranes were blocked with TBST containing 5% non-fat dry milk for 1 h at room temperature, then incubated overnight at 4 °C with primary antibodies. The antibodies used included: VMP1 (1:1000, Affinity, China), GPX4 (1:1000, Affinity, China), FTH1 (1:1000, Affinity, China), LPCAT3 (1:1000, Affinity, China), ACSL4 (1:1000, Affinity, China), Bax (1:2000, Abcam, UK), Bcl-2 (1:2000, Abcam, UK), cleaved caspase-3 (1:1000, CST, USA), cleaved caspase-7 (1:1000, CST, USA), PINK1 (1:1000, CST, USA), Parkin (1:1000, Affinity, China), LC3B (1:1000, CST, USA), p62 (1:1000, CST, USA), collagen II (1:500, Affinity, China), aggrecan (1:500, Abcam, UK), MMP-3 (1:1000, Affinity, China), MMP-13 (1:1000, Affinity, China), and β-actin (1:1000, Affinity, China). After three washes with PBS, membranes were incubated with species-specific horseradish peroxidase-conjugated secondary antibodies for 1 h at room temperature. Protein bands were visualized using an enhanced chemiluminescence (ECL) detection kit (Millipore, USA) and imaged with a Tanon-4800 chemiluminescence imaging system (Shanghai, China). Band intensities were quantified using ImageJ software (NIH, USA).

### Enzyme-linked immunosorbent (ELISA) assay

NPCs were seeded in 96-well plates at a density of approximately 5 × 10^3^ cells/well. Following the indicated treatments, NPCs were lysed using an appropriate lysis buffer, and the resulting supernatants were collected for analysis. The levels of ferric iron, malondialdehyde (MDA), and reduced glutathione (GSH) were quantified using commercial ELISA kits (Westang, Shanghai, China) in accordance with the manufacturer’s protocols.

### Liperfluo staining

Lipid peroxidation, indicative of ferroptosis, was assessed using the fluorescent probe Liperfluo (Mito-bio, Shanghai, China). NPCs were seeded at a density of approximately 3 × 10^4^ cells per well in 12-well plates containing sterile glass slides. Following the designated treatments, cells were maintained in serum-free medium and incubated with 5 µM Liperfluo working solution at 37 °C for 30 min. After incubation, cells were rinsed to remove excess probe and subsequently imaged using a fluorescence microscope.

### Quantification of intracellular ROS levels

ROS were detected using a fluorescent probe (DCFH-DA, Beyotime, China) in accordance with the manufacturer's protocol. NPCs were seeded at a density of approximately 3 × 10^4^ cells per well in 12-well plates containing sterile glass slides. After the designated treatments, NPCs were incubated with 10 μM DCFH-DA diluted in serum-free medium for 30 min at 37 °C in the dark. Upon completion of staining, cells were washed three times with PBS to eliminate excess dye. Fluorescent signals indicative of ROS accumulation were visualized and captured using a fluorescence microscope. The fluorescence intensity was quantified using ImageJ software.

### JC-1 staining

The mitochondrial membrane potential of NPCs was assessed using a JC-1 Mitochondrial Membrane Potential Assay Kit (Beyotime, China). In brief, NPCs were seeded into confocal dishes at a density of approximately 3 × 10^4^ cells per dish and subjected to the appropriate experimental treatments. Following treatment, 1 mL of JC-1 working solution was added to each dish, and the cells were incubated in the dark at 37 °C for 30 min. In polarized mitochondria, JC-1 accumulates as aggregates, emitting red fluorescence, whereas in depolarized mitochondria, it remains in monomeric form, exhibiting green fluorescence. The red-to-green fluorescence intensity ratio was calculated as an index of mitochondrial membrane potential. Nuclear staining was performed using Hoechst 33342. Fluorescence signals were captured using a Zeiss LSM800 laser scanning confocal microscope (Germany), and fluorescence quantification was performed based on the red/green ratio.

### Mitochondrial superoxide assay

Mitochondrial ROS production was measured using the Mito-SOX Red mitochondrial superoxide indicator (Beyotime, China). Briefly, NPCs were seeded into confocal dishes at a density of approximately 3 × 10^4^ cells per dish and subjected to the appropriate experimental treatments. Following treatment, NPCs cultured on coverslips were incubated with 5 μM Mito-SOX for 10 min in the dark, followed by three PBS washes. The cells were then mounted using an anti-fade mounting medium. Fluorescence images were captured using a Zeiss LSM800 laser scanning confocal microscope (Germany), and the intensity of red fluorescence was quantified.

### Measurement of adenosine triphosphate (ATP)

The intracellular ATP levels in chondrocyte samples were measured using an enhanced ATP assay kit (Beyotime, China). Briefly, NPCs cultured in a 6-well plate were lysed with cell lysis buffer, and the lysates were centrifuged to obtain the supernatant. ATP working solution was prepared and used to analyze the samples according to the manufacturer’s instructions. Absorbance was measured at the specified wavelength using a spectrophotometer, and ATP levels in different groups were calculated based on the standard curve.

### Hematoxylin and eosin (HE) staining

Rat intervertebral disc tissues were carefully harvested and fixed in 4% paraformaldehyde. The fixed samples were then decalcified in an ethylenediaminetetraacetic acid (EDTA) solution for approximately 4 to 6 weeks. After decalcification, tissues were subjected to a graded ethanol dehydration series, cleared in xylene, and embedded in paraffin. Paraffin blocks were sectioned and stained with hematoxylin and eosin (HE) using a commercially available staining kit (Solarbio, China).

### Safranin O/fast green staining

Rat intervertebral disc specimens were collected and fixed in 4% paraformaldehyde (PFA), followed by decalcification using a commercial decalcifying reagent (Bestbio, China) until adequate softening of the osseous tissue was achieved. After decalcification, the tissues underwent dehydration through a graded ethanol series and were subsequently embedded in paraffin. Serial sections at a thickness of 5 µm were prepared, deparaffinized in xylene, and rehydrated through descending concentrations of ethanol. Safranin O/fast green staining (Solarbio, China) was performed in accordance with the manufacturer’s protocol.

### Immunohistochemical (IHC) analysis

Paraffin-embedded tissue sections were initially deparaffinized in xylene and rehydrated through a graded ethanol series. Antigen retrieval was performed using a sodium citrate buffer. Immunohistochemical (IHC) staining was conducted using a two-step detection system following the manufacturer’s protocol (ZSGB-BIO, Beijing, China). To reduce nonspecific binding, sections were blocked with 5% bovine serum albumin (BSA) for 30 min at room temperature, followed by overnight incubation at 4 °C with the appropriate primary antibody. The next day, sections were incubated with a goat-derived secondary IgG antibody for 1 h at room temperature and counterstained with hematoxylin for 5 min. The stained tissues were observed using a Leica optical microscope, and high-resolution images were acquired with a digital slide scanner (NanoZoomer S60, Hamamatsu, Japan). Quantitative analysis was carried out on samples obtained from five rats per experimental group.

### Statistical analysis

Data analysis was performed using GraphPad Prism 9.0 (GraphPad Software Inc., USA), with results presented as mean ± standard deviation (SD). For comparisons between two groups, a t-test was used, while differences among multiple groups were assessed using one-way ANOVA followed by Tukey’s post hoc test. All data were derived from at least three independent experiments to ensure reliability. For animal experiments, results were based on repeated trials with a minimum of six samples per group. Statistical significance was considered at *P* < 0.05, with the following designations: *P < 0.05, **P < 0.01, ***P < 0.001, and ****P < 0.0001.

## Results

### VMP1 expression decreases and ferroptosis occurs after IVDD

**Fig. 1:**
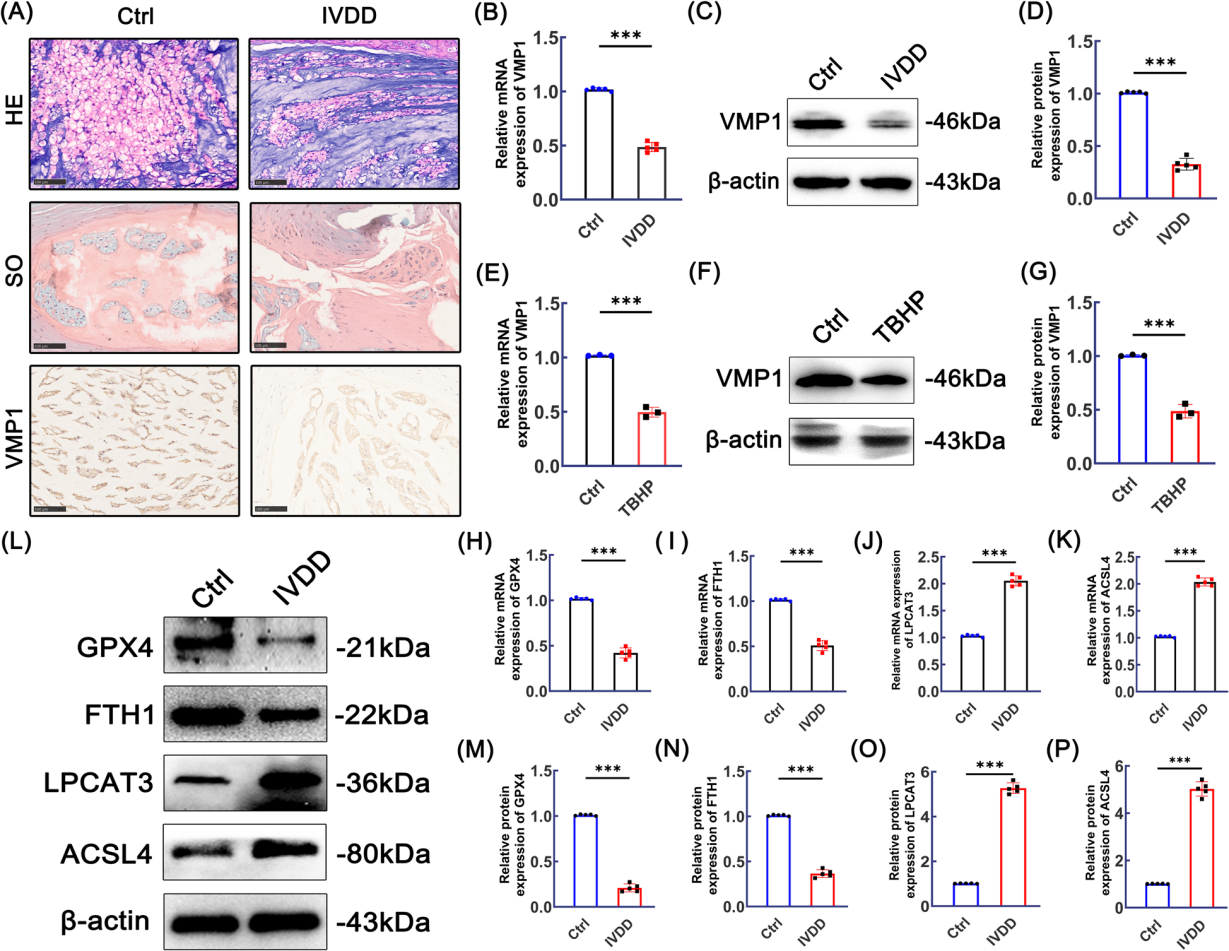
VMP1 expression decreased in IVDD, and ferroptosis occurred. **A **Representative images of H&E staining, Safranin O/Fast Green staining, and VMP1 immunohistochemistry in nucleus pulposus tissue of rats from the control and IVDD groups. The scale bar is set at 100 μm (*n* = 5). **B** VMP1 expression levels in the nucleus pulposus tissue of control and IVDD rats were assessed by qPCR (*n* = 5). **C**-**D** Western blot analysis of VMP1 expression in nucleus pulposus tissue from control and IVDD rats (*n* = 5). **E** The changes in VMP1 expression in NPCs following TBHP treatment were evaluated using qPCR (*n* = 3). **F**-**G** After treatment with TBHP, the protein expression changes of VMP1 in NPCs was determined through western blot analysis and quantification (*n* = 3). **H-K** The expression levels of GPX4, FTH1, LPCAT3 and ACSL4 in nucleus pulposus tissue from control and IVDD rats were assessed using qPCR (*n* = 5). **L-P** The protein expression levels of GPX4, FTH1, LPCAT3 and ACSL4 in nucleus pulposus tissue from control and IVDD rats were determined through western blot analysis and quantification (*n* = 5). The data are presented as mean ± SD, based on three or five separate independent experiments. *p < 0.05, **p < 0.01, ***p < 0.001, ***p < 0.0001, ns for no significance

Based on current research, VMP1 has been implicated in a range of diseases; however, its expression in IVDD has not yet been documented. To investigate the relationship between VMP1 expression and ferroptosis in IVDD, rat and cellular IVDD models were established to enable subsequent validation. The validity of the rat IVDD model was initially confirmed by H&E and Safranin O/Fast Green staining. IHC analysis subsequently revealed a decreased expression of VMP1 in the degenerated nucleus pulposus tissue (Fig. 1A). We further confirmed the changes in VMP1 expression during IVDD progression using PCR analysis, as shown in Fig. 1B. The qPCR results were consistent with the aforementioned IHC findings. Western blot analysis further confirmed that VMP1 expression was reduced in the nucleus pulposus tissue of IVDD model rats (Fig. 1C-D). Subsequently, we further validated the above findings through in vitro experiments. qPCR results showed a significant decrease in VMP1 mRNA expression in NPCs after exposure to TBHP (Fig. 1E). Furthermore, western blot results revealed a similar trend (Fig. 1F-G). Next, we investigated whether ferroptosis plays a role in the process of IVDD. qPCR analysis revealed that the expression levels of GPX4 and FTH1 were significantly decreased, while those of LPCAT3 and ACSL4 were markedly increased in the nucleus pulposus of IVDD model rats (Fig. 1H–K). As shown in Fig. 1L–P, these findings were further confirmed by Western blot analysis.

### VMP1 deficiency exacerbates apoptosis and degeneration in NPCs

**Fig. 2:**
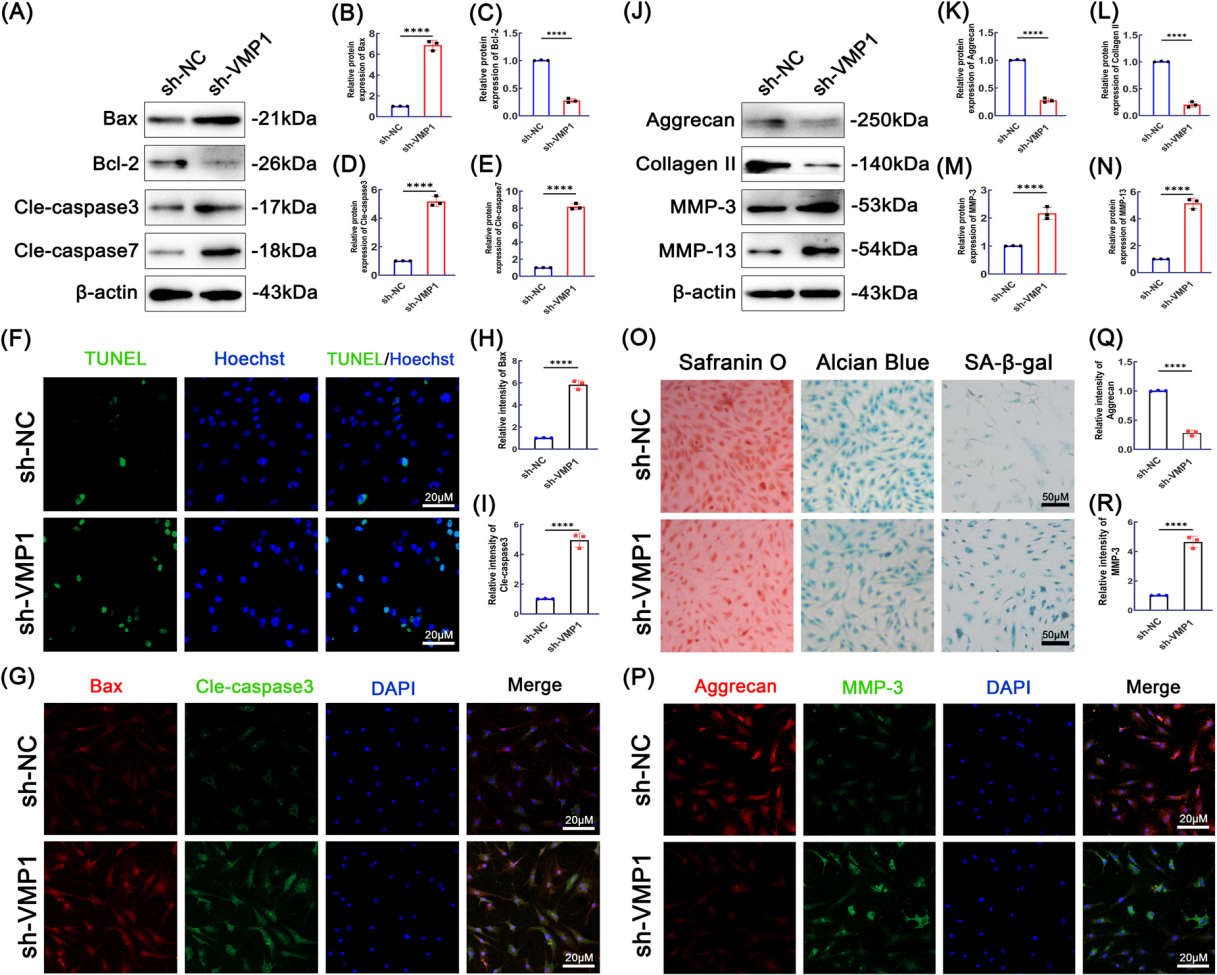
The effects of VMP1 knockdown on apoptosis and degeneration of NPCs. **A**-**E** The expression levels of Bax, Bcl-2, cleaved caspase-3, and cleaved caspase-7 proteins in NPCs across various treatment groups were evaluated using western blot analysis and subsequent quantification (*n* = 3). **F** Cell apoptosis measured by TUNEL staining (scale bar = 20 μm, *n* = 3). **G** The expression changes of Bax and cleaved caspase-3 in NPCs were detected through co-immunofluorescence (scale bar = 20 μm, *n* = 3). **H**-**I** Quantification of fluorescence intensity for Bax and cleaved caspase-3 expression. **J**-**N** The protein expression levels of Aggrecan, collagen II, MMP-3, and MMP-13 in NPCs treated as described above was determined through western blot analysis and quantification (*n* = 3) (**O**) Analysis of NPCs phenotype, glycosaminoglycan synthesis, acidic mucopolysaccharide production, and senescence was performed using Safranin O staining, Alcian blue staining, and SA-β-gal staining (scale bar = 50 μm, *n* = 3). **P**-**R** Co-immunofluorescence was used to detect the changes in expression of Aggrecan and MMP-3 in NPCs (scale bar = 20 μm, *n* = 3). The data represent the mean ± SD values derived from three independent experiments. ns (no significance), *p < 0.05, **p < 0.01, ***p < 0.001 and ***p < 0.0001

To investigate the physiological function of VMP1, NPCs were transfected with a highly efficient and specific shRNA targeting VMP1. The knockdown efficiency was confirmed by assessing protein expression levels (Supplementary Fig. 1A–B, *P* < 0.05). We then examined the impact of VMP1 knockdown (KD) on IVDD by analyzing phenotypic changes, including apoptosis and matrix degeneration. Western blotting was used to quantify the expression of apoptosis-related proteins, including Bax, Bcl-2, cleaved caspase-3, and cleaved caspase-7. Compared to the sh-NC group, VMP1 KD resulted in upregulated expression of Bax, cleaved caspase-3, and cleaved caspase-7, along with a significant downregulation of Bcl-2 (Fig. 2A–E). Apoptosis in NPCs was further evaluated using the TUNEL assay, which showed an increased apoptotic rate following VMP1 KD (Fig. 2F). Co-immunofluorescence analysis of Bax and cleaved caspase-3 corroborated these findings (Fig. 2G–I). In addition, Western blotting was performed to assess the expression of ECM catabolic and anabolic markers, including Aggrecan, collagen II, MMP-3, and MMP-13. As shown in Fig. 2J–N, VMP1 KD significantly decreased Aggrecan and collagen II expression, while increasing MMP-3 and MMP-13 levels compared to the sh-NC group. Further phenotypic assessment by optical microscopy with Safranin O staining demonstrated reduced glycosaminoglycan (GAG) content and accelerated ECM degradation in VMP1 KD cells. Consistently, Alcian blue staining indicated a decline in acidic mucopolysaccharide secretion following VMP1 KD. SA-β-galactosidase staining confirmed that VMP1 KD aggravated cellular senescence in NPCs (Fig. 2O). Immunofluorescence staining for Aggrecan and MMP-3 yielded consistent results (Fig. 2P–R).

### VMP1 deficiency induces ferroptosis in NPCs

**Fig. 3:**
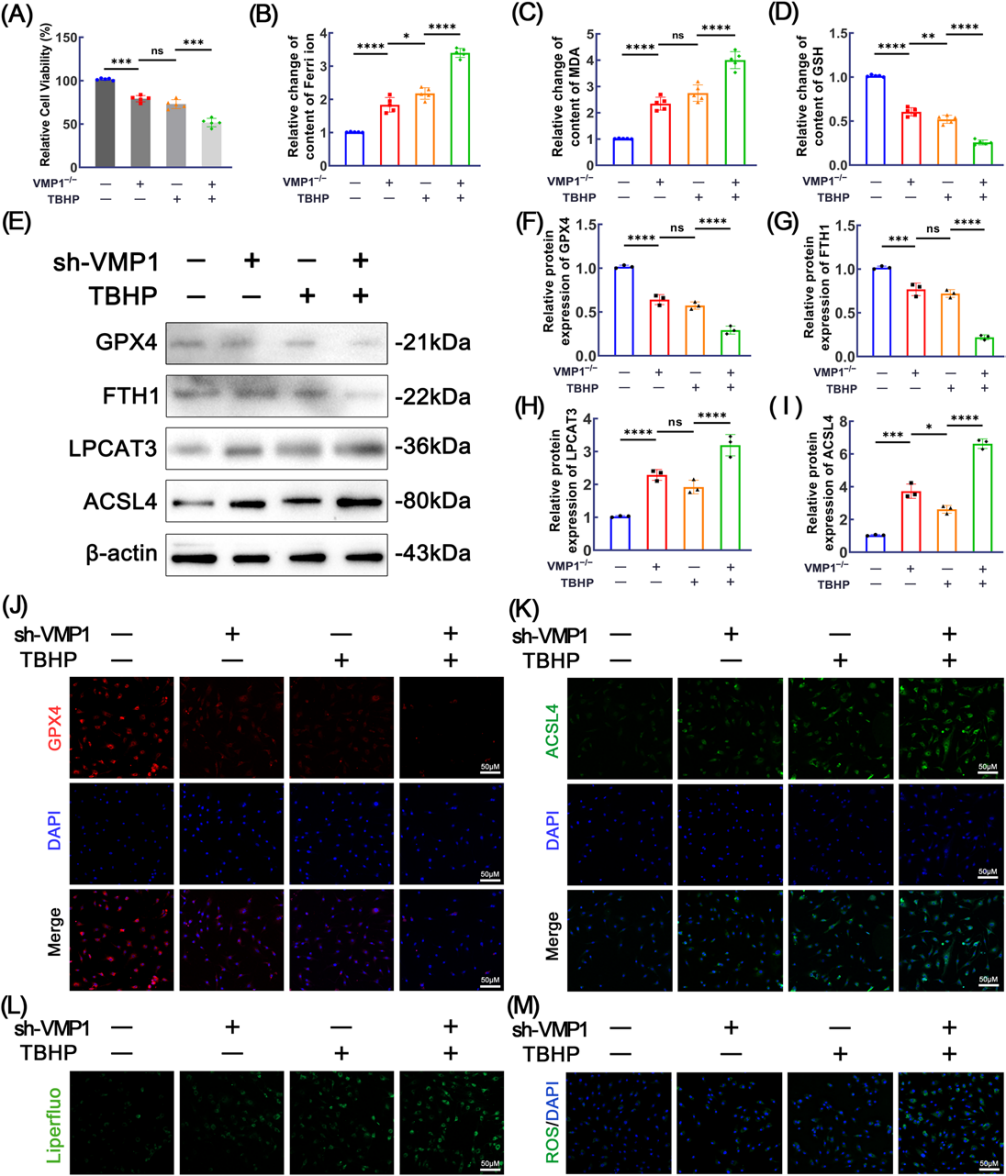
The effects of VMP1 knockdown on the ferroptosis of NPCs. **A** Cell viability across various treatment groups was assessed using the CCK8 assay (*n* = 5). **B** An assay kit was used to measure ferric iron levels in NPCs across different experimental groups (*n* = 5). **C** MDA levels in NPCs from various experimental groups were measured using a specific assay kit (*n* = 5). **D** GSH levels in NPCs across different experimental groups were assessed using a specific assay kit (*n* = 5). **E**-**I** Western blotting and subsequent quantification were performed to evaluate the protein levels of GPX4, FTH1, LPCAT3, and ACSL4 in NPCs across the different groups. **J** Immunofluorescence staining combined with quantitative analysis was employed to assess the expression of GPX4 in NPCs (scale bar = 50 μm, *n* = 3). **K** Immunofluorescence staining, followed by quantitative analysis, was utilized to evaluate ACSL4 expression in NPCs (scale bar = 50 μm, *n* = 3). **L** Liperfluo staining was performed to assess lipid peroxidation levels in NPCs across different groups. The scale bar is set at 50 μm (*n* = 3). (M) ROS staining was performed to assess oxidative stress levels in NPCs across different groups. The scale bar is set at 50 μm (*n* = 3). The data represent the mean ± SD values derived from three independent experiments. *p < 0.05, **p < 0.01, ***p < 0.001, ***p < 0.0001, ns for no significance

Previous studies have demonstrated that TBHP is effective activators of ferroptosis and oxidative stress, and they are commonly used to establish in vitro models of IVDD. The impact of VMP1 KD on ferroptosis was investigated in vitro by administering different treatments to NPCs. The CCK8 assay demonstrated a reduction in cell viability following TBHP treatment, which was further aggravated by VMP1 KD (Fig. 3A). To further investigate the role of VMP1 in regulating ferroptosis in NPCs, we subsequently measured the levels of ferric iron, GSH, and MDA in NPCs across different treatment groups. As shown in Fig. 3B–D, ferric iron and MDA levels were markedly elevated in both the TBHP-treated and VMP1 KD groups compared to controls, with the highest release observed under combined treatment conditions. In contrast, GSH levels were reduced in both the TBHP-treated and VMP1 KD groups, with the most pronounced decrease detected in the combined treatment group. Western blot analysis was conducted to evaluate the expression of ferroptosis-related markers in NPCs, including GPX4, FTH1, LPCAT3, and ACSL4. Compared with the control group, both TBHP stimulation and VMP1 knockdown significantly reduced the expression levels of GPX4 and FTH1, while notably upregulating LPCAT3 and ACSL4. These alterations were most prominent in the combined treatment group (Fig. 3E–I). To validate the involvement of VMP1 in the ferroptosis of NPCs, immunofluorescence was utilized to examine the expression of GPX4 and ACSL4, and consistent results were obtained (Fig. 3J–K). Additionally, Liperfluo staining and ROS staining was used to assess lipid peroxidation and oxidative stress levels in NPCs. The results showed that both TBHP treatment and VMP1 knockdown increased phospholipid peroxidation and ROS levels in NPCs, with a more pronounced elevation observed in the combined treatment group (Fig. 3L–M).

### Effects of VMP1 KD on mitophagy and mitochondrial function in NPCs

**Fig. 4:**
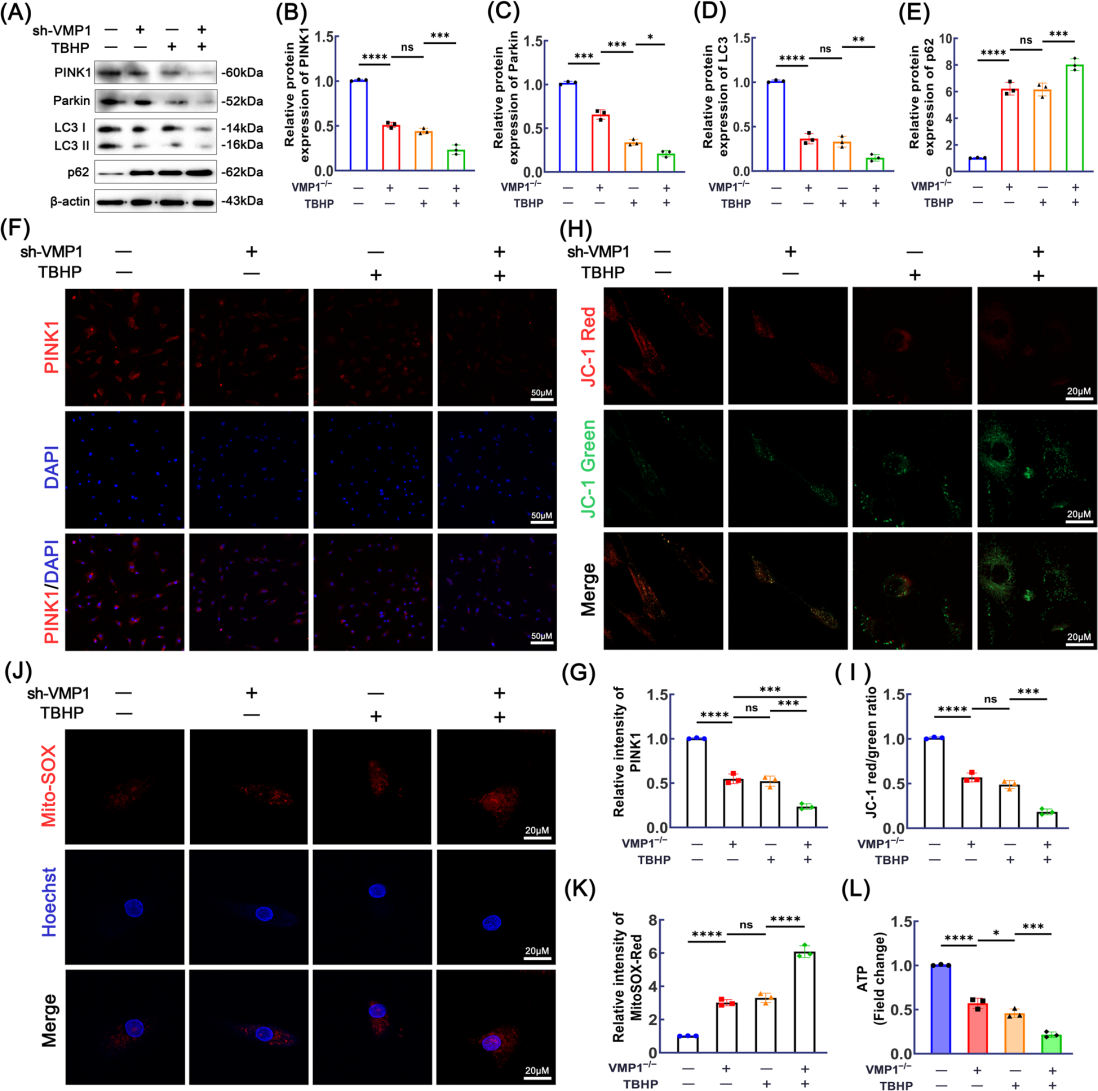
VMP1 KD inhibit mitochondrial autophagy and exacerbate mitochondrial dysfunction in NPCs. **A**-**E** Western blot analysis and quantitative assessment were conducted to determine the protein expression levels of PINK1, Parkin, LC3 and p62 in NPCs from each group. **F**-**G** Immunofluorescence followed by quantitative analysis was utilized to evaluate PINK1 expression levels in NPCs. **H**-**I** The mitochondrial membrane potential in NPCs was analyzed using JC-1 fluorescent probe staining combined with quantitative evaluation. (scale bar, 20 μm, *n* = 3). **J**-**K** Mito-SOX staining was employed to assess the levels of mitochondrial ROS in NPCs. Scale bar, 20 μm (*n* = 3). **L** The ATP production in NPCs was measured using a commercial assay kit (*n* = 5). These findings are presented as the mean ± SD values derived from three independent experiments. ns (no significance), *p < 0.05, **p < 0.01, ***p < 0.001 and ***p < 0.0001

Mitophagy is essential for maintaining energy metabolism and cellular homeostasis in NPCs. To investigate the involvement of VMP1 in mitophagy and homeostasis, we analyzed the expression of key mitophagy markers. As shown in Fig. 4A–E, protein levels of mitophagy-related markers PINK1, Parkin, and LC3 were reduced following TBHP treatment or VMP1 KD, while p62 expression was notably elevated compared to the control group. Interestingly, these alterations were further amplified in the group subjected to the combined interventions. Immunofluorescence analysis of PINK1 expression provided additional confirmation of these findings (Fig. 4F–G). To further investigate the effects of VMP1 on mitochondrial homeostasis and function, we measured the mitochondrial membrane potential using the JC-1 fluorescent probe and assessed mitochondrial function based on the red/green fluorescence ratio. As shown in Fig. 4H–I, the red/green fluorescence ratio of mitochondrial membrane potential was significantly decreased in the TBHP group and the VMP1 KD group compared to the control group. Moreover, the reduction in the red/green fluorescence ratio was more pronounced in the combined treatment group. Subsequently, mitochondrial superoxide production was assessed further with the use of Mito-SOX staining. As anticipated, superoxide levels were elevated after TBHP treatment, and this effect was further intensified by VMP1 KD (Fig. 4 J–K). As the primary energy hubs of cells, mitochondria rely on ATP synthesis, which serves as a key indicator of their functional state. Accordingly, we evaluated mitochondrial function by quantifying ATP production. As shown in Figure 4L, ATP production in NPCs was significantly reduced in the TBHP group and the VMP1 KD group compared to the control group. Furthermore, the reduction in ATP levels was more pronounced in the combined treatment group. In summary, our results demonstrate that VMP1 knockdown impairs mitochondrial autophagy and aggravates mitochondrial dysfunction in NPCs.

### VMP1 alleviates TBHP-induced mitochondrial damage in NPCs by promoting mitochondrial autophagy

**Fig. 5:**
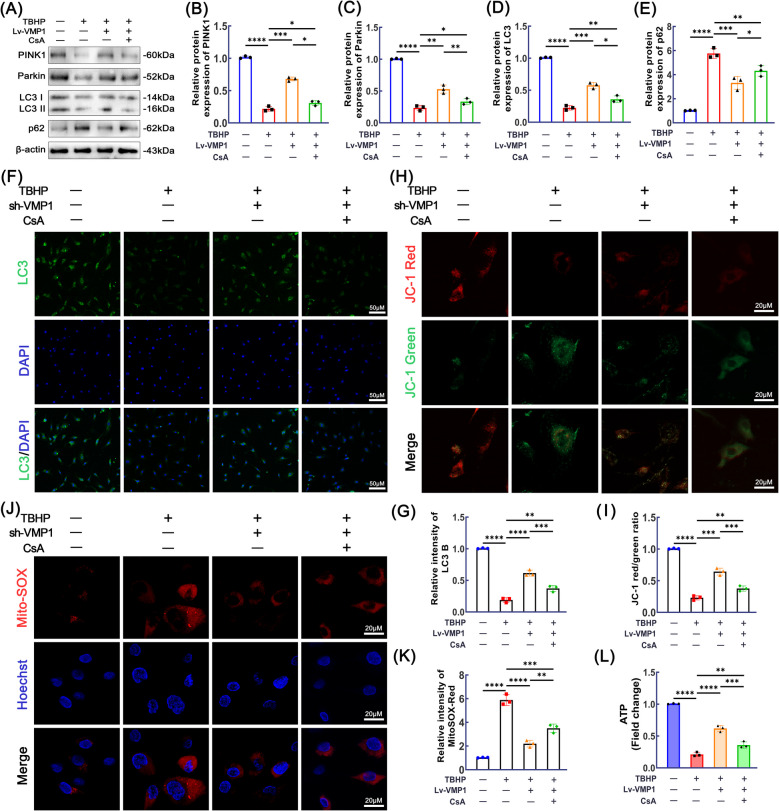
VMP1 alleviated TBHP induced NPCs mitochondria dysfunction via mitophagy. **A**-**E** Western blotting and quantitative analysis were performed to measure the protein expression levels of PINK1, Parkin, LC3, and p62 in NPCs across all groups (*n* = 3). **F**-**G** LC3 expression in NPCs was determined using immunofluorescence. The scale bar is set at 50 μm (*n* = 3). **H**-**I** JC-1 fluorescent probe staining, combined with quantitative analysis, was employed to assess the mitochondrial membrane potential in NPCs (scale bar, 20 μm, *n* = 3). (J-K) Mito-SOX staining was employed to assess the levels of mitochondrial ROS in NPCs (scale bar, 20 μm, *n* = 3). **L** The ATP production in NPCs was measured using a commercial assay kit (*n* = 5). Data are shown as mean ± SD (*n* = 3). One representative result from three independent experiments is shown. ns (no significance), *p < 0.05, **p < 0.01, ***p < 0.001 and ***p < 0.0001

To investigate the specific mechanisms through which VMP1 modulates mitophagy and mitochondrial function, we employed CsA to inhibit the fusion of autophagosomes with lysosomes, effectively blocking autophagy. To evaluate whether VMP1 overexpression (OE) mitigates mitochondrial dysfunction in NPCs, we utilized a recombinant lentiviral vector to induce VMP1 overexpression and confirmed its expression levels through Western blot analysis (Supplementary Fig. 1C–D, *P* < 0.05). The data presented in Fig. 5A–E show that, in comparison to the TBHP group, VMP1 overexpression (OE) markedly enhanced the expression of PINK1, Parkin, and LC3 II/I, while decreasing p62 levels, indicating that VMP1 OE facilitates PINK1/Parkin-mediated mitophagy. However, the use of CsA reversed the promoting effect of VMP1 on mitochondrial autophagy. Immunofluorescence analysis of PINK1 expression further validated these findings (Fig. 5F–G). Furthermore, JC-1 fluorescent probe staining demonstrated that, compared to TBHP treatment, VMP1 OE significantly enhanced the red/green fluorescence ratio. However, this ratio was significantly reduced after CsA pretreatment (Fig. 5H–I). To further investigate mitochondrial superoxide production, Mito-SOX staining was performed. As anticipated, ROS levels were elevated after TBHP treatment, while VMP1 OE reduced mitochondrial ROS generation. In contrast, CsA treatment worsened mitochondrial ROS production (Figs. 5J–K). As shown in Fig. 5L, compared to TBHP treatment, VMP1 OE significantly increased ATP production in NPCs, while CsA partially reversed the restorative effect of VMP1 OE on ATP generation.

### VMP1 alleviates ferroptosis in NPCs through the activation of mitophagy

**Fig. 6:**
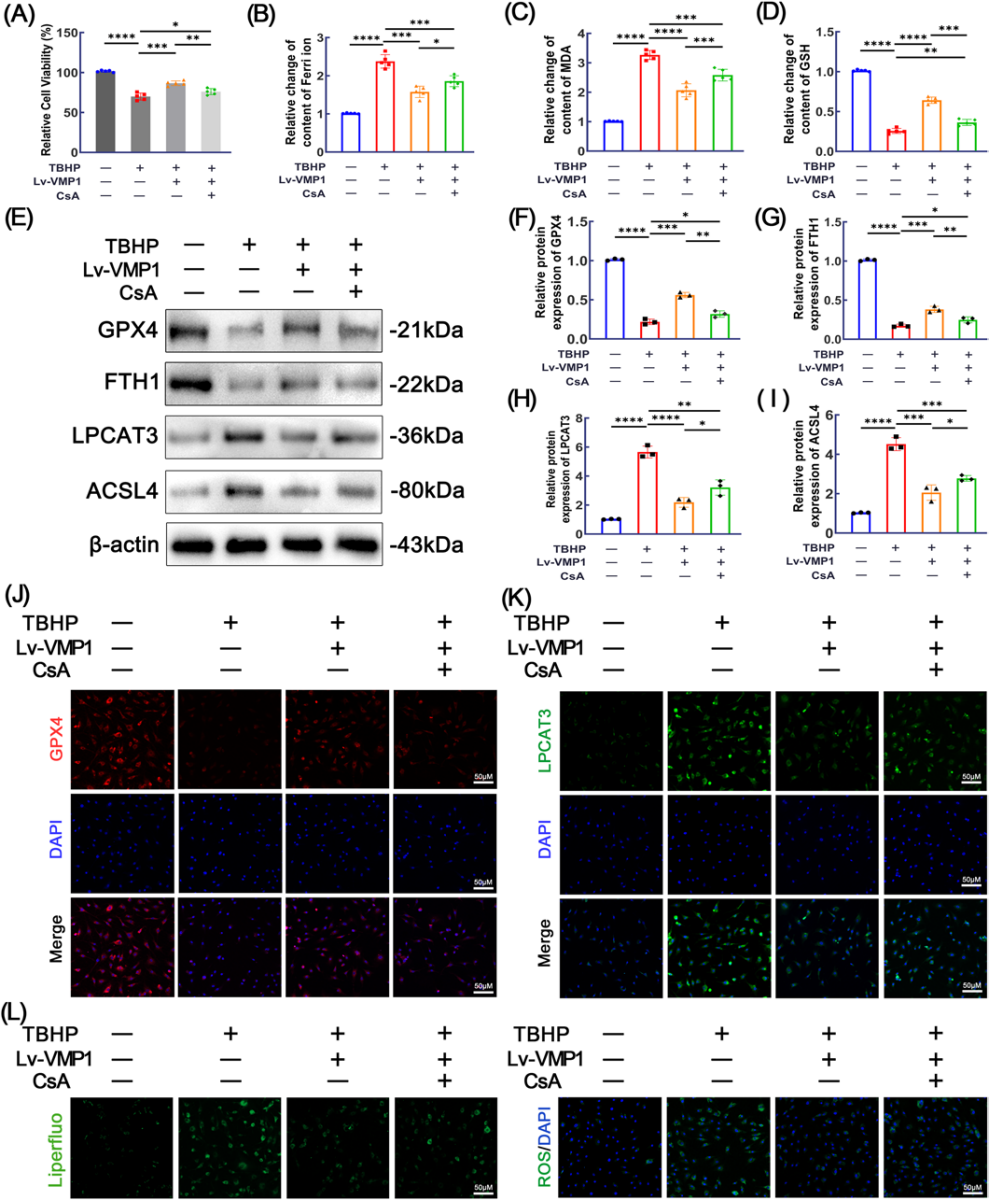
VMP1 reduces ferroptosis in NPCs through the activation of mitophagy. **A** Cell viability in different treatment groups was evaluated using the CCK-8 assay (*n* = 5). **B** Ferric iron levels in NPCs were quantified using a commercial assay kit across various experimental conditions (*n* = 5). **C** MDA concentrations in NPCs were measured using a dedicated assay kit under each treatment condition (*n* = 5). **D** GSH content in NPCs was assessed with a specific assay kit across different groups (*n* = 5). **E**-**I** Western blot analysis and densitometric quantification were conducted to determine the expression levels of GPX4, FTH1, LPCAT3, and ACSL4 in NPCs across groups. **J** Immunofluorescence staining with quantitative analysis was used to evaluate GPX4 expression in NPCs (scale bar = 50 μm, *n* = 3). **K** LPCAT3 expression in NPCs was assessed by immunofluorescence staining and quantification (scale bar = 50 μm, *n* = 3). **L** Liperfluo staining was performed to examine lipid peroxidation levels in NPCs under different treatments (scale bar = 50 μm, *n* = 3).(M) ROS levels in NPCs were detected by ROS staining across the treatment groups (scale bar = 50 μm, *n* = 3). Data are presented as mean ± SD from three independent experiments. *p < 0.05, **p < 0.01, ***p < 0.001, ****p < 0.0001, with ns indicating no significance

We further explored the role of VMP1 overexpression in the regulation of ferroptosis and its underlying molecular mechanisms. The CCK-8 assay demonstrated that VMP1 OE restored the TBHP-induced reduction in NPC viability, whereas CsA pretreatment exacerbated cellular damage (Fig. 6A). To further investigate the potential mechanisms by which VMP1 regulates ferroptosis in NPCs, we measured the levels of ferric iron, GSH, and MDA across different treatment groups. As shown in Fig. 6B–D, VMP1 OE attenuated the TBHP-induced increases in ferric iron and MDA levels while restoring GSH levels. In contrast, CsA pretreatment elevated ferric iron and MDA levels and further decreased GSH levels. To explore whether VMP1 inhibits ferroptosis through the promotion of mitophagy, we performed western blot analysis. As expected, compared with the TBHP-treated group, VMP1 overexpression restored the expression of GPX4 and FTH1 while downregulating LPCAT3 and ACSL4. In contrast, CsA pretreatment reversed the upregulation of GPX4 and FTH1 and the downregulation of LPCAT3 and ACSL4 (Fig. 6E–I). These findings were further corroborated by immunofluorescence analysis of GPX4 and ACSL4, as shown in Fig. 6J–K. In addition, Liperfluo and ROS staining were performed to evaluate lipid peroxidation and oxidative stress levels in NPCs. The results showed that, compared with TBHP treatment, VMP1 overexpression significantly reduced phospholipid peroxidation and ROS levels in NPCs, whereas CsA treatment reversed these effects (Fig. 6L–M).

## Discussion

IVDD is a chronic, degenerative, and disabling condition, and is recognized as a leading cause of chronic LBP [37]. Due to the complexity of its pathogenesis and the unclear underlying molecular mechanisms, current clinical interventions primarily focus on symptom management rather than disease modification. In this study, we found that VMP1 expression was significantly downregulated in IVDD, concomitant with increased ferroptosis. KD of VMP1 exacerbated apoptosis of NPCs and degradation of the ECM. Furthermore, VMP1 KD promoted oxidative stress-mediated ferroptosis in NPCs, inhibited mitophagy, and aggravated mitochondrial dysfunction. Based on these findings, we propose that stabilizing or enhancing VMP1 expression may represent a novel therapeutic approach for IVDD. In addition, we identified that PINK1/Parkin-mediated mitophagy is the central mechanism through which VMP1 ameliorates mitochondrial dysfunction and suppresses ferroptosis. To the best of our knowledge, this is the first study to uncover the therapeutic potential of VMP1 in the context of IVDD. Therefore, VMP1 may serve as a promising target for therapeutic intervention in IVDD.

The exact pathogenesis of IVDD remains poorly understood. Thus, unraveling the precise mechanisms underlying IVDD and developing targeted molecular therapies offer significant potential for future research and clinical advancements. The key pathological features of IVDD include chronic inflammation, elevated oxidative stress, disruption of mitochondrial homeostasis, degradation of the ECM, and multiple forms of PCD [38, 39]. These interconnected events not only exacerbate disc degeneration but also converge to aggravate mitochondrial injury, thereby establishing a vicious cycle that accelerates cellular dysfunction and disease progression [40]. Mitophagy, a selective form of autophagy, is essential for preserving mitochondrial integrity and function by removing damaged or dysfunctional mitochondria. This process helps to limit the accumulation of ROS and suppress the initiation of cell death pathways [41]. Among the regulatory mechanisms, the PINK1/Parkin axis is widely recognized as the canonical pathway mediating mitophagy. Nevertheless, existing research on NPCs has predominantly examined individual pathological factors in isolation, with limited insight into the crosstalk and interplay among these processes. VMP1 is an integral membrane protein that plays a pivotal role in cellular processes, particularly by participating in autophagosome formation and its subsequent fusion with lysosomes, thereby enhancing autophagic flux [26]. The regulatory role of VMP1 in the pathogenesis of various diseases, including neurodegenerative disorders, hepatitis, cancer, and pancreatitis, has been well established [30–32, 42]. In our current study, we confirmed the downregulation of VMP1 expression in degenerated NP following IVDD, which, as expected, was accompanied by the occurrence of ferroptosis in NPCs.

Previous studies have demonstrated that NP degeneration directly contributes to IVDD and that various forms of PCD are involved in the pathological transformation of NPCs [43]. Ferroptosis, an iron-dependent form of PCD that has garnered increasing attention, has been implicated in the pathogenesis of multiple diseases, including IVDD. In aging or degenerative IVDD, disrupted iron homeostasis results in intracellular iron accumulation, initiating the Fenton reaction and excessive ROS generation [12, 44]. These events lead to lipid peroxidation and mitochondrial dysfunction, culminating in GPX4 inactivation, GSH depletion, and ultimately ferroptotic cell death [36]. This ferroptotic cascade amplifies inflammatory responses, promotes apoptosis and ECM degradation, thereby impairing NPC function and accelerating IVDD progression [45]. Although ferroptosis has been recognized as a contributing factor to IVDD, studies elucidating its underlying mechanisms and therapeutic potential remain limited. In the present study, we established an oxidative stress-induced ferroptosis model in HNPCs using TBHP. NPCs subjected to VMP1 knockdown or TBHP treatment exhibited marked oxidative stress–induced ferroptosis, with a synergistic enhancement observed upon combined treatment. Notably, prior to ferroptosis onset, VMP1 deficiency was found to aggravate apoptosis and compromise ECM synthesis. These findings strongly support the central role of oxidative stress–driven ferroptosis in NPC dysfunction and IVDD pathogenesis, and suggest that stabilizing or enhancing VMP1 expression may represent a novel and promising therapeutic strategy.

Mitochondria function as the cell’s energy powerhouse and are critical for preserving cellular homeostasis and mediating responses to various stress signals. As the primary site of reactive ROS and free radical production, mitochondria are central to the regulation of oxidative stress and ferroptosis. Mitochondrial dysfunction is implicated in cellular injury and multiple degenerative diseases, with several studies highlighting its role in IVDD [46, 47]. Mitophagy, by selectively degrading damaged or dysfunctional mitochondria, maintains mitochondrial quality and prevents the buildup of defective organelles, thereby limiting ROS accumulation [48]. Importantly, distinct cell types exhibit unique metabolic profiles and iron homeostasis mechanisms. Consequently, the role of mitophagy in ferroptosis may be dualistic and highly dependent on specific cellular contexts and stress conditions [49]. Recent studies have reported divergent roles of mitophagy in the regulation of ferroptosis. PINK1/Parkin-mediated mitophagy has been shown to promote ferroptosis in hepatocytes by degrading GPX4 localized to mitochondria [50]. Excessive activation of mitophagy can lead to the lysosomal degradation of large numbers of mitochondria, resulting in the release of free fatty acids and increased lipid peroxidation, thereby facilitating ferroptosis [51]. Conversely, other findings indicate that mitophagy can inhibit ferroptosis by removing damaged mitochondria and decreasing mitochondrial ROS production [52]. Notably, receptor-mediated mitophagy has been reported to prevent ferroptosis through the suppression of mitochondrial ROS. Furthermore, targeting AMPK/PINK1/Parkin-mediated mitophagy has been shown to reduce ferroptosis both in vitro and in vivo, offering protective effects against kidney stone formation [53]. Additional studies suggest that mitophagy activation can attenuate chondrocyte ferroptosis and slow osteoarthritis progression [54]. Therefore, VMP1-mediated mitophagy may serve as a therapeutic strategy to suppress ferroptosis and reduce ROS levels in NPCs. As expected, VMP1 knockdown or TBHP treatment inhibited mitophagy and exacerbated mitochondrial dysfunction. Conversely, VMP1 overexpression promoted PINK1/Parkin-mediated mitophagy, thereby alleviating mitochondrial impairment and suppressing ferroptosis. However, this protective effect was reversed by the mitophagy inhibitor CsA. This study elucidates the interplay between mitophagy and ferroptosis in the context of IVDD.

## Conclusion

This study is the first to demonstrate that VMP1 expression is downregulated following IVDD and is associated with the occurrence of ferroptosis. VMP1 deficiency exacerbates NPCs apoptosis and ECM degradation. Furthermore, the absence of VMP1 leads to the activation of ferroptosis, suppression of mitophagy, and disruption of mitochondrial function. By promoting PINK1/Parkin-dependent mitophagy, VMP1 restores mitochondrial function and inhibits oxidative stress–driven ferroptosis in NPCs. Thus, VMP1 holds promise as a potential therapeutic target for mitigating IVDD.

## Supplementary Information

Supplementary Material 1: Supplementary Figure 1. Results of Supplementary Information. (A-B) Western blot analysis and quantification of VMP1 were performed on HNP cells transfected with sh-VMP1. (C-D) The transfection efficiency of Lv-VMP1 in cells 72 hours after transduction at a MOI of 100. The expression of VMP1 after transfection was analyzed by western blot. The data represent the mean ± SD values derived from three independent experiments, and were analyzed using *Mann-Whitney U test*. *P < 0.05 vs. negative control group, **P < 0.05 vs. positive control group.

## Data Availability

Data is available on request from the authors.
