# Supplementary figures and images for: VMP1 attenuates ferroptosis and mitochondrial dysfunction in nucleus pulposus cells through the PINK1/Parkin-mediated mitophagy pathway
Source: J Orthop Surg Res. 2025 Jul 8;20:630. doi: 10.1186/s13018-025-06033-2 (PMC12239386; doi:10.1186/s13018-025-06033-2)

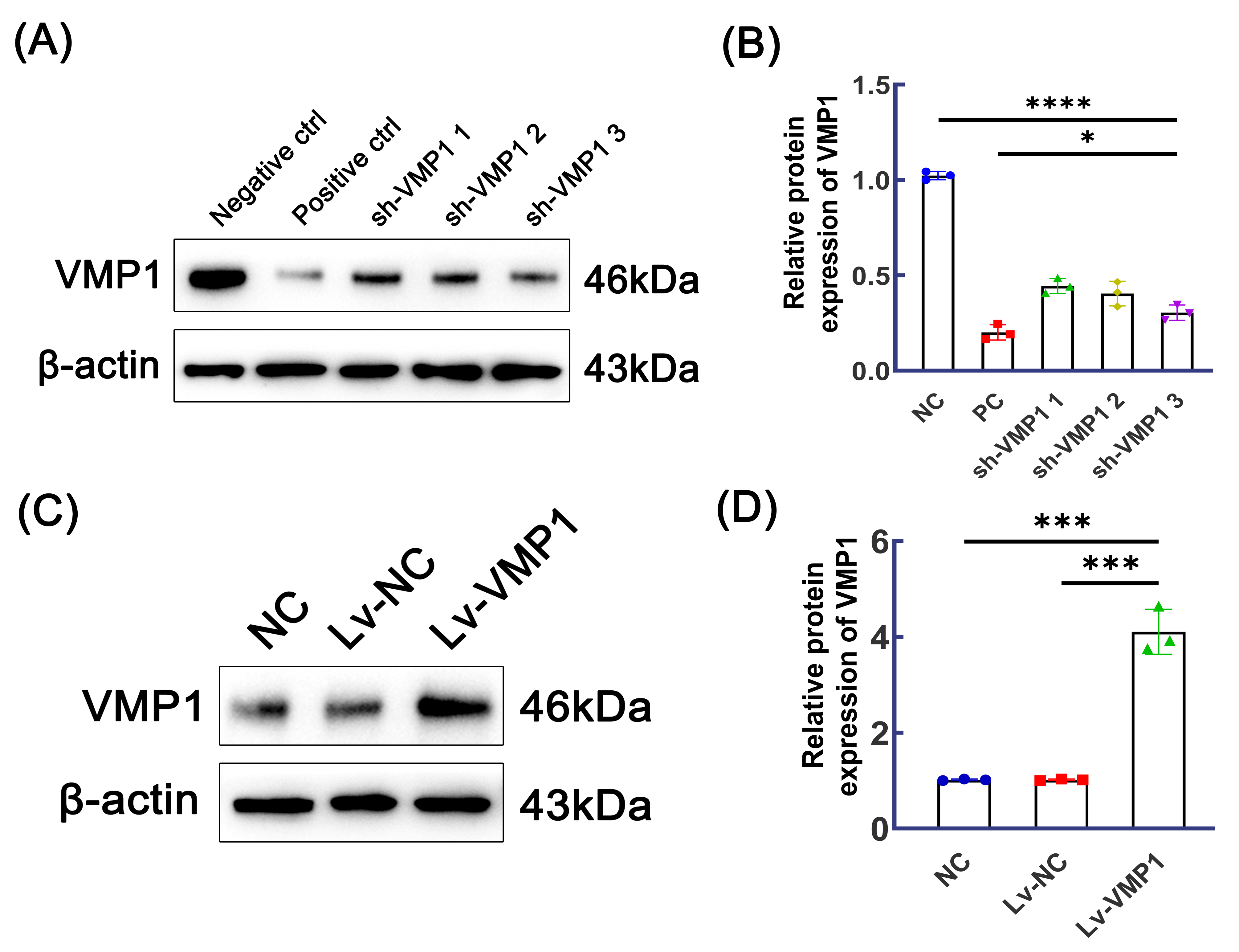

Supplement: Supplementary file 1 — Supplementary Material 1: Supplementary Figure 1. Results of Supplementary Information. (A-B) Western blot analysis and quantification of VMP1 were performed on HNP cells transfected with sh-VMP1. (C-D) The transfection efficiency of Lv-VMP1 in cells 72 hours after transduction at a MOI of 100. The expression of VMP1 after transfection was analyzed by western blot. The data represent the mean ± SD values derived from three independent experiments, and were analyzed using Mann-Whitney U test. *P < 0.05 vs. negative control group, **P < 0.05 vs. positive control group. [file 13018_2025_6033_MOESM1_ESM.tif]
